# Introduction to the Academy for Healthcare Improvement conference on advancing the methods for healthcare quality improvement research

**DOI:** 10.1186/1748-5908-8-S1-I1

**Published:** 2013-04-19

**Authors:** Theodore Speroff, Paul V Miles, Denise Dougherty, Brian S Mittman, Mark E Splaine

**Affiliations:** 1 Center for Health Services Research, Geriatric Research, Education, and Clinical Center (GRECC), Veterans Affairs Tennessee Valley Healthcare System, Nashville, Tennessee, 37212, USA; 2 Vanderbilt University School of Medicine, Nashville, Tennessee, 37232, USA; 3 American Board of Pediatrics, Chapel Hill, North Carolina, 27514, USA; 4 Agency for Healthcare Research and Quality, Rockville, Maryland, 20850, USA; 5 VA HSR&D Center for the Study of Healthcare Provider Behavior, Veterans Affairs Greater Los Angeles Healthcare System, Sepulveda, California, 91343, USA; 6 Geisel School of Medicine at Dartmouth, Hanover, New Hampshire, 03755, USA

## 

Nearly two decades ago, the International Scientific Symposium on Improving the Quality and Value of Health Care was created to foster the mission of building a scholarly foundation for quality improvement education and research. The program of presentations at the inaugural symposium was comprised almost entirely of non-experimental, before-after studies that are subject to significant problems with reliability and validity. The passing years have seen an evolving trend toward robust study designs with more rigorous statistical methods such as process control and interrupted time-series analyses. The foundation for quality improvement established over the past twenty years has created a repository of evidence. Quality improvement has a proliferation of publications appearing in peer-reviewed journals. Discipline specific outlets have emerged such as the British Medical Journal of Quality and Safety in Health Care, Implementation Science, Joint Commission Journal on Quality and Patient Safety, Journal for Healthcare Quality, and Journal of Nursing Care Quality. However, the methodology in quality improvement and implementation science must become on par with the methods in health economics and related areas of health services research if quality improvement research is to materialize into evidence-based health care and contribute to health care reform.

Practitioners and researchers in the field of quality improvement recognize the need to elevate the relevance and value of their research. The potential contribution of implementation science as a strategic approach for obtaining safe, efficient, and effective health care is too easily overlooked without a solid record of publication in the scientific literature. The need to generate the best evidence for systematic quality improvement was the driving factor for a conference on Advancing the Methods for Healthcare Quality Improvement Research, held on May 7-8, 2012 in Arlington, Virginia. The aims of the conference were 1) to reflect on the past two decades of quality improvement and quality improvement research and appreciate the emergence of methods and techniques in the literature today, and 2) to define quality improvement research, characterize its strengths, and propose future directions for quality improvement research and implementation science.

This conference set out to further the goal of elevating quality improvement research to a higher level of validity and value. The conference presented information on the conduct of quality improvement research, including study design, data registries, comparative effectiveness, and the issue of health care disparities. In addition, there were several talks on sophisticated methods including robust quasi-experimental designs, hybrid research designs, and risk-adjusted statistical process control. By the use of key speakers, a call for abstracts for podium and poster presentations, and case-based learning using examples of best methods in the literature, this conference was a forum on the current state and future needs of quality improvement research and its methodological and technical issues.

The proceedings of this conference are presented in this supplement to provide a series of concise papers that describe the key messages of the presentations and a commentary about the contribution to advancing the field of quality improvement and implementation science. Session one was a set of three talks on the architecture of study design for doing quality improvement research presented by Dr. Duncan Neuhauser, Dr. Mark Bauer, and Dr. Peter Margolis. Session two comprised two talks on the implementation of a practice-based learning registry for quality improvement (Dr. Richard Colletti) and advanced statistical process control methods for reporting findings from quality surveillance registries (Dr. Michael Matheny). Sessions three and four introduced new frontiers for quality improvement research in comparative effectiveness (Dr. David Atkins) disparities research (Dr. Donald Goldmann), implementation science (Dr. John Ovretveit), and the contribution to government contract initiatives (Dr. Joanne Lynn). In session five, Dr. Brian Mittman delivered an eloquent presentation on what the future holds for quality improvement research. Sessions six and seven concluded the conference with audience roundtable discussions on evaluating strengths and weaknesses of quality improvement research using two examples of best practices and making recommendations for the future direction of quality improvement and research.

Our objective for this program was to formulate the types of questions relevant to advancing quality improvement and implementation science and their appropriate study designs. Our overall goal is to bring about more effective application of quality improvement science in health care delivery and to learn new areas for application of quality improvement research. With this conference proceeding, we disseminate the highlights. Voice-over recordings of the full presentations are available at the Academy for Healthcare Improvement web site (http://www.A4HI.org) as well as a listing of recommended readings. By sharing the knowledge gained from this conference, we hope to inspire the spirit of initiative, innovation, motivation, and thinking that fuels the passion for improving health and delivery of health care.

